# Two New Species of *Pentacarinus* from the Acrotiarini Tribe in Burmese Amber (Hemiptera, Fulgoromorpha, Cixiidae)

**DOI:** 10.3390/insects15060439

**Published:** 2024-06-11

**Authors:** Keyi Deng, Feiyang Liang, Thierry Bourgoin, Menglin Wang

**Affiliations:** 1 Key Laboratory of Southwest China Wildlife Resources Conservation, Ministry of Education, China West Normal University, Nanchong 637009, China; dky525129@163.com; 2 College of Life Sciences, China West Normal University, Nanchong 637009, China; 3 Key Laboratory of Economic Crops Genetic Improvement and Integrated Utilization, Hunan University of Science and Technology, Xiangtan 411201, China; feiyang_sco@foxmail.com; 4 Institut de Systématique, Evolution, Biodiversité, ISYEB-UMR 7205 MNHN-CNRS-Sorbonne, Université-EPHE-University Antilles, Muséum National d’Histoire Naturelle, CP 50, 57 rue Cuvier, 75005 Paris, France; thierry.bourgoin@mnhn.fr

**Keywords:** taxonomy, paleoentomology, Cenomanian, planthopper

## Abstract

**Simple Summary:**

An increasing number of fossil specimens of planthoppers from Burmese amber are being described. Few of them belong to extant families. Here, we described two new fossil species of *Pentacarinus*: *P. maculosus* sp. nov. and *P. tenebrosus* sp. nov. in the tribe Acrotiarini in the extant family Cixiidae, from Kachin state of northern Myanmar. A revised diagnosis for the genus and a key to species identification are also provided.

**Abstract:**

Two new species, *Pentacarinus maculosus* sp. nov. and *Pentacarinus tenebrosus* sp. nov., from Burmese amber are described. Alongside the type species *P. kachinensis* already described, they are easily distinguishable by the tegmina patterns. The diagnosis of the genus *Pentacarinus* is amended, notably with fusion of Pcu + A_1_ distad of forking CuA, the fork ScP + R approximately close to basal 1/5 of tegmen, basad of forking CuA, and only one transverse veinlet *ir* between RP and RA on forewings. Additionally, a key to these three species of *Pentacarinus* is provided.

## 1. Introduction

In the past ten years, Burmese amber inclusions have attracted significant attention, leading to the description of an increasing number of fossil taxa [1]. Up to the end of the year 2023, 2805 species have been documented in Burmese amber [2], including 45 species in planthoppers [2]. They contribute to an already rich register of fossil planthoppers, which were mainly documented from Germany and Brazil (reviewed in [3]), but also in locations such as Lebanon [4], Spain [5] and Argentina [6], among others. Although local taxonomic diversity may appear to emphasize selected lineages, potentially biasing our general understanding of planthopper evolution dynamics, Burmese amber inclusions offer a valuable insight into their past diversification. This is particularly the case for the family Cixiidae Spinola, 1839, which currently holds the highest number of identified fossils among planthoppers in extant families from Burmese amber [7].

Cixiidae taxon was formally erected as Cixioides subfamily, in family Fulgorites by Spinola [8], then later upgraded to family level by Schaum [9]. It is the most diversified group in planthoppers, including over 250 genera and 2640 species at present, distributed all around the world [10]. Larval stages are usually found underground feeding on roots [11,12], while adult stages have been documented hosted on a great variety of plants from more than 51 orders of Monilophytes (Polypodiales, Cyatheales, etc.), Gymnosperms (Pinales), Monocots (Asparagales, Poales, etc.) and Eudicots, on which they widely predominate on Asterales, Rosales, Fabales and Myrtales [10]. Cixiidae with its sister group of Delphacidae Leach, 1815 constitute of the superfamily Delphacoidea Leach, 1815 [13,14].

Fossil taxa in Cixiidae encompass 36 genera and 47 species [10] representing, respectively, 14.4% and 1.8% of the taxonomic diversity of the family. They are reported from seven tribes: Cixiini Spinola, 1839 [15,16,17,18,19], Eucarpiini Emeljanov, 2002 [17], Mnemosynini Emeljanov, 1992 [17,20,21], Pentastirini Emeljanov, 1971 [17,22], Pintaliini Metcalf, 1938 [17], Bothriocerini Muir, 1923 [17,23], and the exclusively fossil tribe Acrotiarini Bourgoin & Luo, 2021 [24,25]. Among them, the tribe with the most diversity fossil taxa is Cixiini, followed by Mnemosynini and Acrotiarini [10].

Luo et al. [24] erected the tribe Acrotiarini to include the following three genera: *Acrotiara* Bourgoin & Luo, 2021 (type species: *A. multigranulata* Luo & Bourgoin, 2021), *Delphitiara* Bourgoin & Luo, 2021 (type species: *D. tibiocoronata* Luo & Bourgoin, 2021) and *Pentacarinus* Bourgoin & Luo, 2021 (type species: *P. kachinensis* Luo & Bourgoin, 2021) from Burmese amber. A fourth genus, *Maculixius* Bourgoin & Wang, 2022 (type species: *M. jiewenae* Wang & Bourgoin, 2022), was described the year after [25] from the same location.

According to Luo et al. [24], the fossil tribe Acrotiarini were left unplaced in the pentastirinian lineage of the Cixiidae. They can be easily distinguished from other cixiids by the following unique combination of characters: a pentacarinated mesonotum; presence of paired sublaterofrontal carinae; and forewing venation with an arched RA with two or three terminals delimitating anteriorly a C1 cell submedially wider than apically [24]. All taxa exhibit MP with four terminals, and CuA with two terminals on the forewing [24,25]. In this study, two new species are described, namely, *P. maculosus* sp. nov. and *P. tenebrosus* sp. nov. We also provide the revised diagnosis of the genus *Pentacarinus* and the key to the species of this genus.

## 2. Materials and Methods

The specimens were collected in Hukawng Valley in Tanai Township, Kachin State of northern Myanmar, which deposit is dated 98.79 ± 0.62 Ma in the Cenomanian period of the mid-Cretaceous [26]. After polishing with three types of grinding paste in turn (coarse, fine and very fine), specimens were studied using an Olympus SZX7 stereomicroscope for observation, and a Leica M205FA stereomicroscope with a Leica MC190 HD camera equipped with software LAS X version 2017.2.0 for photography. Line drawings were illustrated with software CorelDRAW 2021 to complete the general framework and software SAI2 for details. The type specimens are deposited in the College of Life Sciences, China West Normal University, Nanchong, Sichuan, China.

The terminologies of forewing venation follow Luo et al. [24] and Bourgoin et al. [27], hindwing and female terminalia follow Luo et al. [24]. The metatibiotarsal formula (s–t)/tI/tII corresponds to the number of lateral spines (s) on the metatibia, the number of apical teeth (t) on the metatibia, the number of apical teeth (tI) on metatarsomere I, and the number of apical teeth (tII) on metatarsomere II [7].

## 3. Systematic Paleontology

Order: Hemiptera Linnaeus, 1758

Suborder: Fulgoromorpha Evans, 1946

Superfamily: Delphacoidea Leach, 1815

Family: Cixiidae Spinola, 1839

Tribe: Acrotiarini Bourgoin & Luo, 2021 (in pentastirinian lineage sec. Luo et al. [24])

 

Genus: *Pentacarinus* Bourgoin & Luo, 2021

Type species: *Pentacarinus kachinensis* Luo & Bourgoin, 2021

 

**Amended diagnosis:** Vertex wider at base than length at middle, anterior margin angulate; frons with sublateral carinae distinctly visible in dorsal view in their dorsal part, almost reaching median ocellus ventrally; pedicel of antenna elongated oval, just surpassing the lateral margin of compound eyes; postclypeus with median carina; tegmina with anterior margin regularly curved until distal extremity between RP and MP_1_; veins on forewings with setae arranged in a ‘V’ pattern, tubercles more or less prominent arranged in pairs on either side of the vein; ScP + R common stem as long as basal cell with forking of ScP + RA and RP in first 1/5 of the tegmina, basad to forking of CuA (fisrt 1/4) and basad to fusion of Pcu + A_1_; one transverse veinlet *ir* between RA and RP; hindwing with RP, MP and CuA respectively with 2, 2 and 3 terminals of V-type [12]; pronotum narrow; mesonotum pentacarinated with lateral carinae S-shaped, anteriorly concave then convex posteriorly, and sublateral carinae almost joining median carina anteriorly; metatibia without lateral spines, metatarsomere I and II apical teeth with platellae; metatibiotarsal formula: 0–(5–6)/(6–7)/(7–8).

 

Key to *Pentacarinus* species:

1. 1. Tegmen translucent, with only one dark spot in pterostigmal area (Figure 8A in Luo et al. [24]), common stem MP_1+2_ absent (Figure 8C in Luo et al. [24])………………………*Pentacarinus kachinensis* Luo & Bourgoin, 2021

- Tegmen with large brown suffusion, common stem MP_1+2_ short but distinct…………………………………………………………………………………………………………………………2

2. Tegmen with a colorless band in its first basal half; vertex triangular, with anterior margin strongly acute……………………………………………………………………*Pentacarinus maculosus* Deng & Wang, sp. nov.

- Tegmen entirely dark brown in its first basal half; vertex quadrangular, with anterior margin shallowly acute……………………………………………………………………*Pentacarinus tenebrosus* Deng & Wang, sp. nov.

 

*Pentacarinus maculosus* Deng & Wang, sp. nov.

LSIDurn:lsid:zoobank.org:act:67B547C5-A002-4882-9AC2-D8B98A63F1DF

 

**Diagnosis.** Similar to *P. kachinensis*, from which it differs notably by the following characteristics: (1) the presence of a colorless band in first basal half (transparent proximally in *P. kachinensis* and entirely dark brown in *P. tenebrosus* sp. nov.); (2) stem MP_1+2_ short but obviously present (also present in *P. tenebrosus* sp. nov. but almost absent in *P. kachinensis*); (3) vertex more strongly acute anteriorly with distinct long areolets-like areas on each side of vertex (absent in *P. kachinensis* and *P. tenebrosus* sp. nov.).

**Etymology.** The name refers to the colorless patches in the tegmina.

**Type material.** Holotype, female adult (MDHP206), in Burmese amber, from Hukawng Valley (Tanai location), Kachin State, Northern Myanmar.

**Description:**

**Figure 1:**
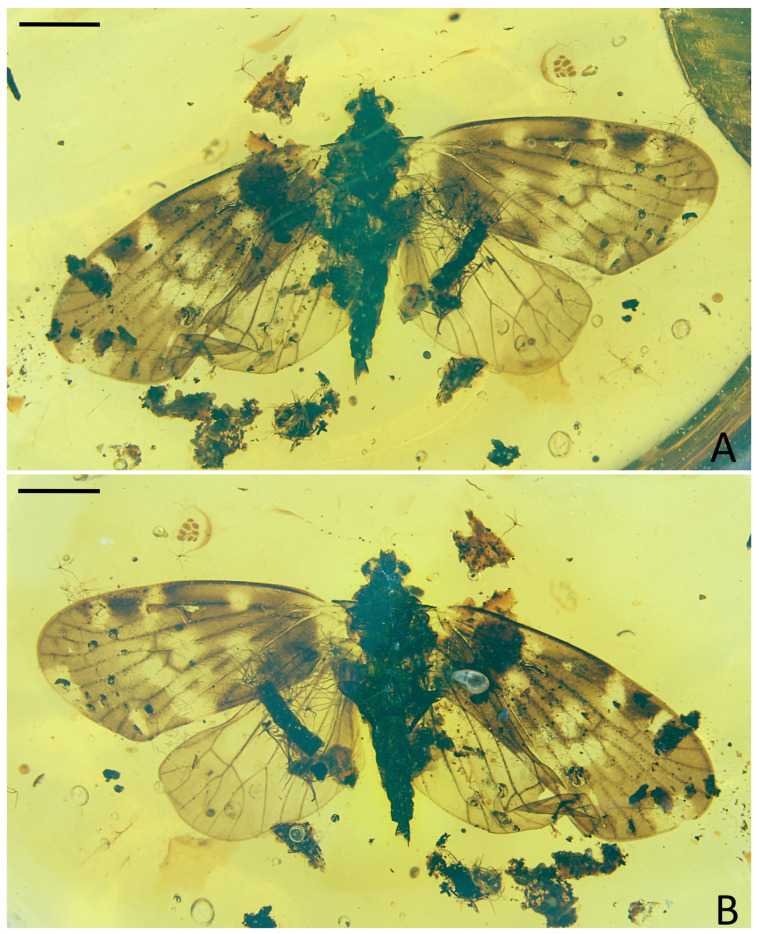
*Pentacarinus maculosus* sp. nov., holotype. (**A**) Adult, dorsal view; (**B**) adult, ventral view. Scale bar: 1 mm.

**Figure 2:**
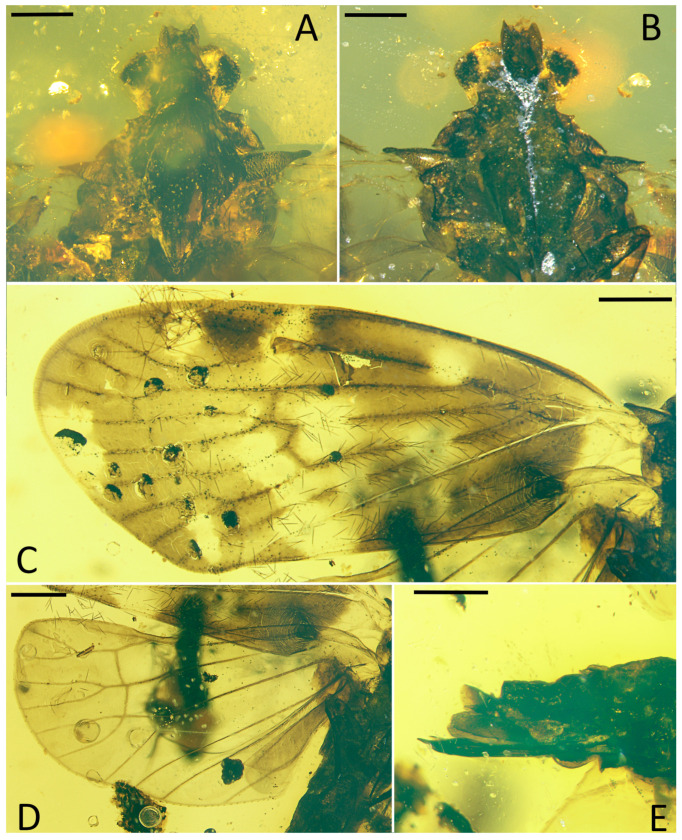
*Pentacarinus maculosus* sp. nov., holotype. (**A**) Head and thorax, dorsal view; (**B**) head, ventral view; (**C**) forewing; (**D**) hindwing; (**E**) female terminalia. Scale bar: 0.3 mm in (**A**,**B**,**E**), 0.5 mm in (**C**,**D**).

**Figure 3:**
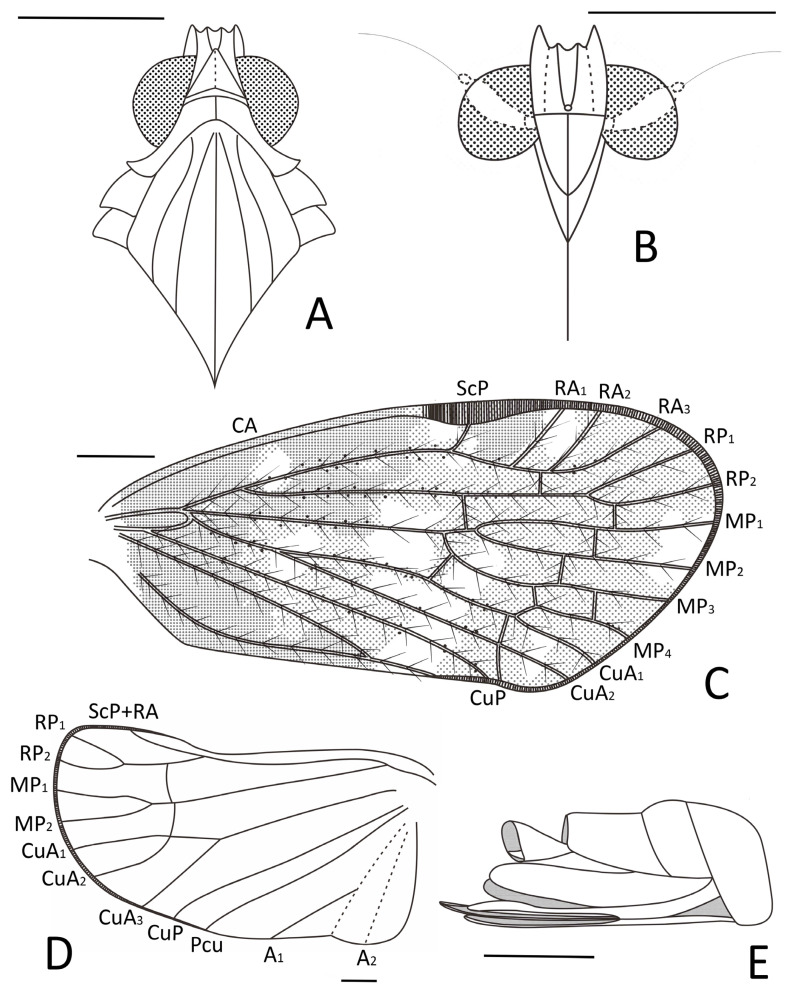
*Pentacarinus maculosus* sp. nov., holotype. (**A**) Head and thorax, dorsal view; (**B**) head, ventral view; (**C**) forewing venation; (**D**) hindwing venation; (**E**) female terminalia. Scale bar: 0.5 mm in (**A**,**B**,**C**), 0.3 mm in (**D**,**E**).

Small size insect (Figure 1, Figure 2 and Figure 3). Total length including tegmina 4.88 mm. The specimen (Figure 1) slightly damaged and deformed; head capsule partly obscured by impurities in dorsal view and antennae missing; fore and right middle legs missing, only left middle leg completed; hind legs with apex of tibia and tarsus missing; the forewings and hindwings unfolded, and well visible.

**Head.** Head width with compound eyes 0.62 mm (Figure 2A). Vertex length 0.13 mm in midline, width at base 0.23 mm (Figure 2A). Compound eyes length 0.36 mm, width 0.22 mm (Figure 2A). Frons length 0.18 mm, width in anterior margin 0.15 mm, width at middle 0.20 mm, width at base 0.19 mm (Figure 2B). Clypeus length 0.34 mm (Figure 2B). Rostrum 0.87 mm (Figure 1B).

**Thorax.** Pronotum length 0.09 mm, width 0.77 mm (Figure 2A). Mesonotum length 0.91 mm, widest width 0.66 mm (Figure 2A).

**Forewings.** (Figure 1A,B, Figure 2C and Figure 3C) Tegmen length 4.19 mm in longest part, width 1.89 mm in widest part; tegmen with an irregular brown pattern covering almost all of tegmen, with one band of colorless patches at 1/4 length of the tegmen at ScP + R and CuA forkings and in claval sector, other colorless areas respectively located in radial area, under pterostigma area, medial area along apical margin, anterior cubital area, and a large broad colorless band in medial area of tegmen extends to the claval margin.

**Hindwings.** Hindwings translucent, length 3.40 mm in longest part, width 1.58 mm in widest part (Figure 2D).

**Legs.** Middle leg femur 0.64 mm, tibia 0.86 mm; hind leg femur 0.57 mm, tibia 0.82 mm without apex (Figure 1B).

**Female terminalia.** (Figure 2E and Figure 3E) Gonapophysis VIII 0.74 mm, falciform, apex obtuse, shorter than gonapophysis IX; gonapophysis IX 0.80 mm, with the acute end, covered with gonapophysis VIII; gonoplacs 0.58 mm, shorter and wider; anal tube 0.17 mm.

 

*Pentacarinus tenebrosus* Deng & Wang, sp. nov.

LSIDurn:lsid:zoobank.org:act:A3702973-CBBD-4BE3-AC19-CE19C86D27FC

 

**Diagnosis.** Similar with *P. kachinensis* and *P. maculosus* sp. nov., but this species differs from them by the following characteristics: (1) tegmen with extensive brown suffusion in first basal half (proximally translucid in *P. kachinensis* and with a band of colorless patches in first 1/4 of the tegmen in *P. maculosus* sp. nov.); (2) metatibiotarsal formula: 0–(5–6)/7/7 (metatibiotarsal formula: 0–6/6/8 in *P. kachinensis*).

**Etymology.** The name refers to the general dark coloration of the tegmina.

**Type material.** Holotype, female adult (MDHP173), in Burmese amber, from Hukawng Valley (Tanai location), Kachin State, Northern Myanmar. Paratype, female adult (MDHP185), in Burmese amber from the same location.

**Description:**

**Figure 4:**
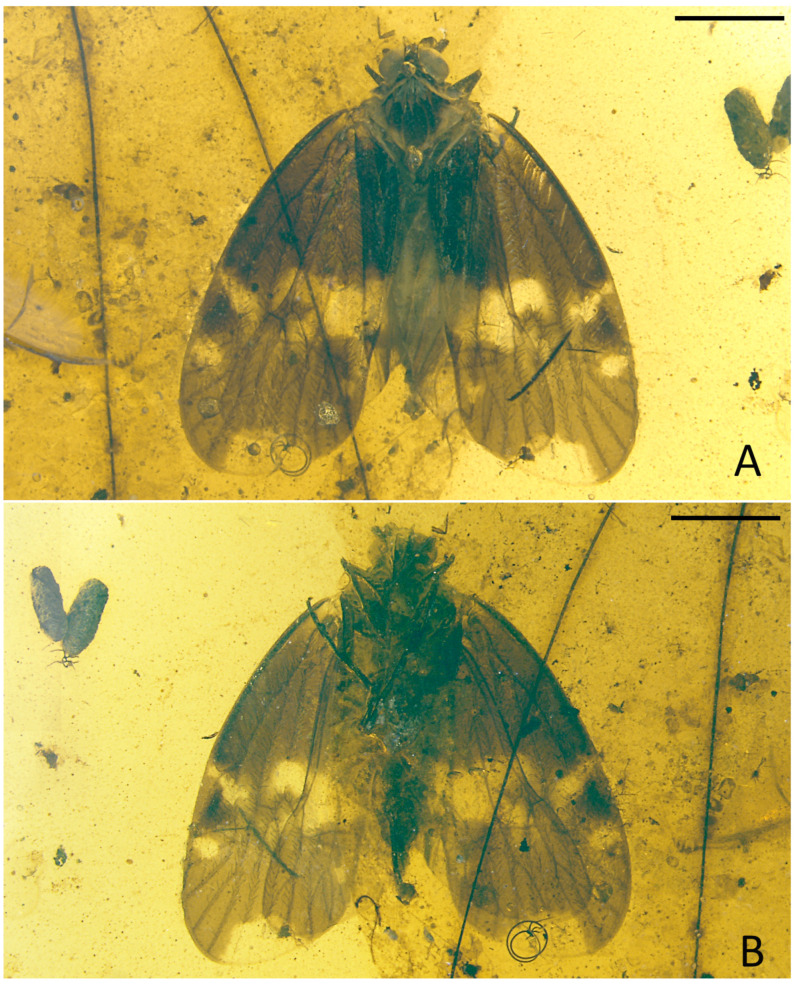
*Pentacarinus tenebrosus* sp. nov., holotype. (**A**) Adult, dorsal view; (**B**) adult, ventral view. Scale bar: 1 mm.

**Figure 5:**
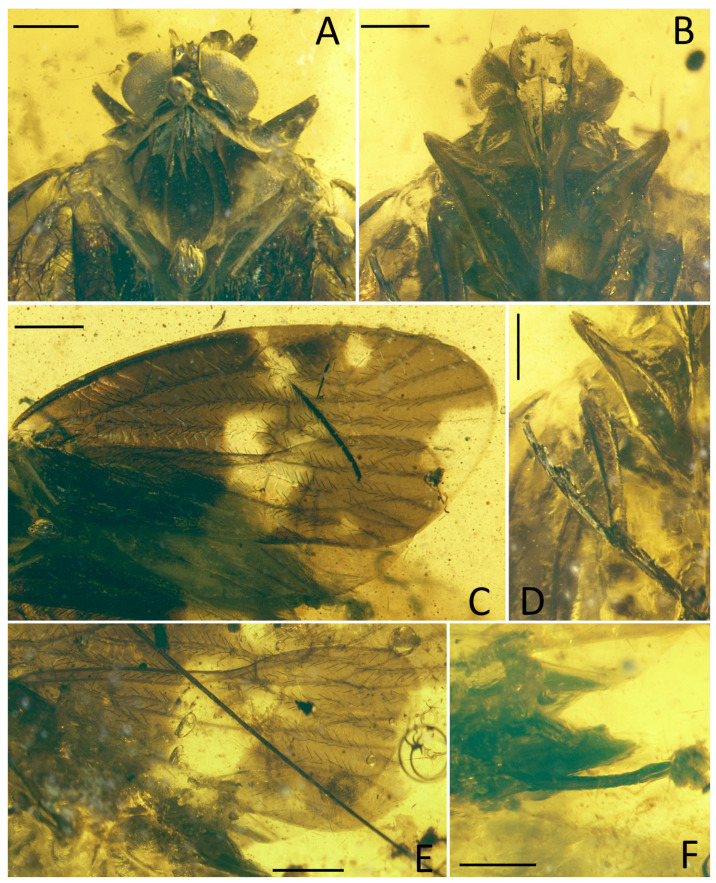
*Pentacarinus tenebrosus* sp. nov., holotype. (**A**) Head and thorax, dorsal view; (**B**) head, ventral view; (**C**) forewing; (**D**) right legs; (**E**) hindwing; (**F**) female terminalia. Scale bar: 0.3 mm in (**A**,**B**,**D**,**F**), 0.5 mm in (**C**,**E**).

**Figure 6:**
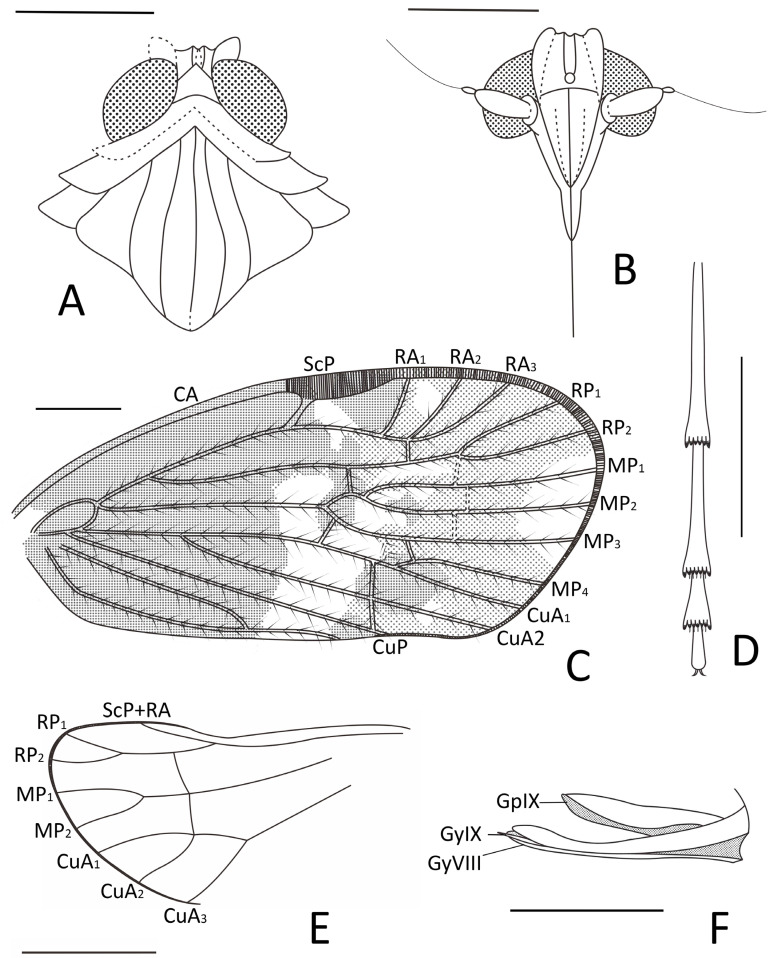
*Pentacarinus tenebrosus* sp. nov., holotype. (**A**) Head and thorax, dorsal view; (**B**) head, ventral view; (**C**) forewing venation; (**D**) apical part of hind leg; (**E**) apical portion of hindwing venation; (**F**) female terminalia. Scale bar: 0.5 mm in (**A**,**B**,**C**,**D**,**F**), 1 mm in (**E**).

**Figure 7:**
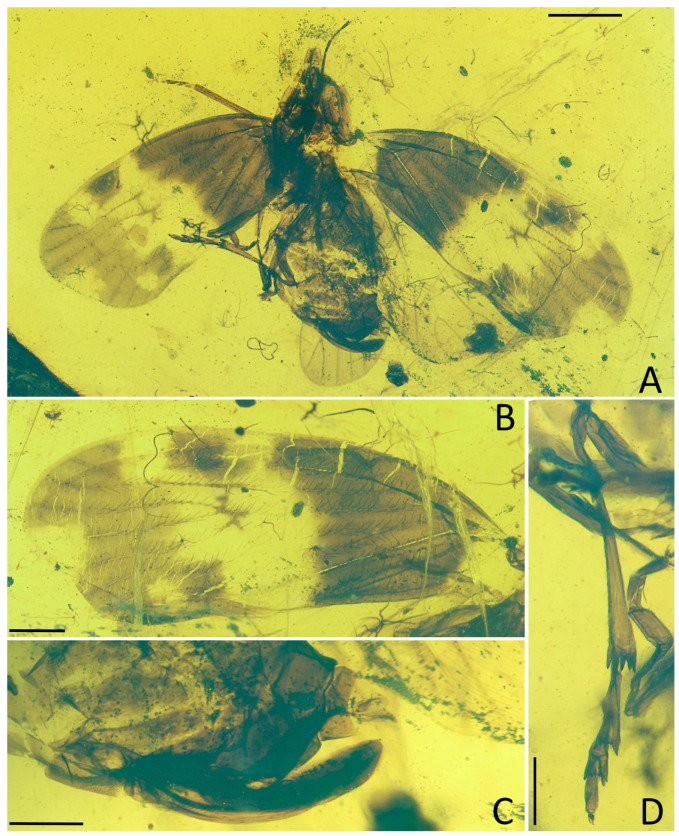
*Pentacarinus tenebrosus* sp. nov., paratype. (**A**) Adult, ventral view; (**B**) forewing; (**C**) female terminalia; (**D**) hind tibia and tarsus. Scale bar: 1 mm in (**A**), 0.5 mm in (**B**), 0.3 mm in (**C**,**D**).

Small size insect (Figure 4, Figure 5, Figure 6 and Figure 7) with total length including tegmina of 4.33 mm. The holotype specimen is well preserved; the forewings and hindwings are folded, hindwing venations are unclear except for the apical portion; fore and middle legs are incomplete and the left ones difficult to observe due to impurities of amber. The paratype is also well preserved, with forewings and hindwings open, but head and thorax were deformed during the fossilization process.

**Head.** Head width with compound eyes 0.64 mm (Figure 5A). Vertex length 0.17 mm in midline, width in anterior margin 0.12 mm, width at middle 0.13 mm, width at base 0.20 mm (Figure 5A). Compound eyes length 0.36 mm, width 0.20 mm (Figure 5A). Antennae pedicel length 0.19 mm, flagellum length 0.35 mm (Figure 6B). Frons length 0.20 mm, width in anterior margin 0.20 mm, width at middle 0.27 mm, width at base 0.25 mm (Figure 5B). Clypeus length 0.45 mm (Figure 5B). Rostrum 0.91 mm (Figure 4B).

**Thorax.** Pronotum length 0.10 mm, width 0.88 mm (Figure 5A). Mesonotum length 0.76 mm, widest width 0.83 mm (Figure 5A).

**Forewings.** (Figure 4A,B, Figure 5C, Figure 6C and Figure 7B) Tegmen length 3.64 mm in longest part, width 1.58 mm in widest part; tegmen with a wide irregular brown pattern covering almost all the tegmen, darker at base than distal portion, small colorless areas respectively located in radial area, medial area along apical margin, under pterostigma area, anterior cubital area and a broad transverse band in median and cubital sectors reaching the anal sector; veins brown.

**Hindwings.** (Figure 4B, Figure 5E, Figure 6E and Figure 7A) Hindwings translucent, the length of hindwings surpassing the body.

**Legs.** (Figure 5D, Figure 6D and Figure 7D) Hind tarsus length 0.61 mm; basitarsomere length 0.36 mm, tarsomere II length 0.13 mm, tarsomere III length 0.12 mm; lateral metatibial spine absent, metatibia with five or six apical teeth, basitarsomere with seven apical teeth, tarsomere II with seven apical teeth; outermost teeth slightly longer; both tarsomere I and II with subapical platellae; metatibiotarsal formula: 0–(5–6)/7/7.

**Female terminalia.** (Figure 5F, Figure 6F and Figure 7C) Similar with *P. maculosus* sp. nov., gonapophysis VIII (GyVIII) 0.73 mm, sickle shaped, long but shorter than gonapophysis IX (GyIX), and the base wider with a triangular hollow; gonapophysis IX only clear at the acute end; gonoplacs (GpIX) 0.53 mm, short and wide, ventral margin slightly curved.

## 4. Discussion and Conclusions

Up to date, the Acrotiarini tribe of Cixiidae encompasses four genera and six species including the two new species in current study. However, this merely represents the initial stage of studying Burmese amber fossils, as numerous specimens, particularly among the Cixiidae, await description. Their study is inherently intricate, as the morphological characters distinguishing major groups in Cixiidae are both scarce and subtle, compounded by the challenge of interpreting them due to the considerable homoplasy observed in the limited morphological traits available at a supra-generic level [28]. Moreover, at the species level, the analysis of male genitalia is most often necessary for contemporary cixiid species. However, such data are not accessible for fossils, leaving only external characters, particularly forewing venation and coloration patterns, as the sole available traits. It is noteworthy that these traits are prone to intraspecific variation. Accordingly, we do not exclude the possibility that *P. maculosus* and *P. tenebrosus* may constitute color morphs of the same species, and they could potentially be synonymized in the future. However, the identification of two specimens of *P. tenebrosus* exhibiting the same overall color pattern but lacking the basal band of colorless patches characteristic of *P. maculosus* provides support for maintaining the distinction between two separate species, as proposed in this paper.

## Data Availability

All relevant data are available from the text and figures.
